# Implementation of grain mapping by diffraction contrast tomography on a conventional laboratory tomography setup with various detectors

**DOI:** 10.1107/S1600576723003874

**Published:** 2023-05-31

**Authors:** Haixing Fang, Wolfgang Ludwig, Pierre Lhuissier

**Affiliations:** a Université Grenoble Alpes, Grenoble INP, CNRS SIMaP, 1130 Rue de la Piscine, 38402 Saint Martin d’Hères, France; b European Synchrotron Radiation Facility, 71 Avenue des Martyrs, 38000 Grenoble, France; c Université de Lyon, INSA Lyon, CNRS MATEIS, 69621 Villeurbanne, France; Ecole National Supérieure des Mines, Saint-Etienne, France

**Keywords:** diffraction contrast tomography, grain reconstruction, CCD detectors, flat panel detectors, synchrotron X-ray diffraction

## Abstract

Laboratory-based diffraction contrast tomography (LabDCT) is a novel technique used to resolve grain orientations and shapes in three dimensions at the micrometre scale using laboratory X-ray sources, allowing the user to overcome the constraint of limited access to synchrotron facilities. To foster the development of this technique, the implementation of LabDCT is illustrated in detail using a conventional laboratory-based X-ray tomography setup, and it is shown that such implementation is possible with the two most common types of detectors: CCD and flat panel. As a benchmark, LabDCT projections were acquired on an AlCu alloy sample using the two types of detectors at different exposure times. Grain maps were subsequently reconstructed using the open-source grain reconstruction method reported in the authors’ previous work. To characterize the detection limit and the spatial resolution for the current implementation, the reconstructed LabDCT grain maps were compared with the map obtained from a synchrotron measurement, which is considered as ground truth. The results show that the final grain maps from measurements by the CCD and flat panel detector are similar and show comparable quality, while the CCD gives a much better contrast-to-noise ratio than the flat panel. The analysis of the grain maps reconstructed from measurements with different exposure times suggests that a grain map of comparable quality could be obtained in less than 1 h total acquisition time without a significant loss of grain reconstruction quality and indicates a clear potential for time-lapse LabDCT experiments. The current implementation is suggested to promote the generic use of the LabDCT technique for grain mapping on conventional tomography setups.

## Abstract 2

Implementation of grain mapping by diffraction contrast tomography has been demonstrated on a conventional tomography setup with two common detectors (CCD and flat panel). Typical grain mapping performance has been characterized for the related experimental conditions and setup, and grain reconstructions from diffraction images acquired with different exposure times demonstrate the possibility of fast grain mapping with laboratory-based X-rays.

## 1. Introduction

Grain mapping techniques have seen a rapid development in the past 25 years to resolve 3D grain orientations and shapes in bulk materials at micrometre and nanometre scales with a particular interest in using X-rays (Poulsen, 2020 ▸). Such techniques have been well established at synchrotron facilities and have led to numerous discoveries and new understandings in materials science (*e.g.* Offerman *et al.*, 2002 ▸; Schmidt *et al.*, 2004 ▸; King *et al.*, 2008 ▸; Simons *et al.*, 2018 ▸; Bhattacharya *et al.*, 2021 ▸). Typical examples include near-field and far-field imaging techniques such as three-dimensional X-ray diffraction microscopy [3DXRD (Poulsen, 2004 ▸; Suter *et al.*, 2006 ▸; Bernier *et al.*, 2011 ▸)] and diffraction contrast tomography [DCT (Ludwig *et al.*, 2008 ▸, 2009 ▸)] with a spatial resolution down to 1 µm, and raster scanning techniques such as differential aperture X-ray microscopy (Larson *et al.*, 2002 ▸) and scanning 3DXRD (Hayashi *et al.*, 2019 ▸; Henningsson *et al.*, 2020 ▸) with a spatial resolution down to ∼200 nm. Another more recent technique is X-ray dark field microscopy (Simons *et al.*, 2015 ▸; Poulsen *et al.*, 2017 ▸; Jakobsen *et al.*, 2019 ▸), which provides very high spatial (∼100 nm) and orientation resolutions (∼0.005°) and is able to map individual dislocation lines. All these techniques, however, require the use of synchrotron radiation, which thus places a serious limitation on access.

To overcome this limitation and broaden the use of grain mapping techniques, laboratory-based X-ray diffraction contrast tomography (LabDCT), adapted from synchrotron DCT, has been developed (King *et al.*, 2013 ▸, 2014 ▸) and commercialized (McDonald *et al.*, 2015 ▸, 2017 ▸). These approaches share the characteristics of using a conical polychromatic beam, confined by an aperture, to illuminate a 3D sample volume and placing a 2D detector behind the sample to record a series of diffraction projections during a stepwise 360° rotation around a vertical axis, while the direct transmitted beam is blocked by a beamstop placed in front of the detector to enhance the diffraction signals. The very first approach (King *et al.*, 2013 ▸) was implemented on a conventional tomography setup with a magnified geometry (sample-to-detector distance is larger than sample-to-source distance, *L*_sd_ > *L*_ss_) using a flat panel detector with a pixel size of 127 µm. The grain indexing was based on Friedel pair matching, and subsequent grain shape reconstruction was performed using algebraic reconstruction techniques, based on iterative forward and back projections. This approach, however, can only deal with a moderate number of grains in the illuminated sample volume to avoid overlap of diffraction spots. The commercial approach, based on a forward modeling strategy, was implemented on Zeiss Xradia setups as an additional modality, using either a Laue focusing geometry (*L*_sd_ = *L*_ss_) with a high-resolution CCD detector (*e.g*. Zeiss Xradia 520 Versa) or a magnified geometry with a flat panel [*e.g.* Zeiss Xradia CrystalCT (Bachmann *et al.*, 2019 ▸; Oddershede *et al.*, 2022 ▸)]. The commercial software *GrainMapper3D* developed by XnovoTech and based on forward projection (Bachmann *et al.*, 2019 ▸) offers an easy-to-use and robust method for grain reconstruction. However, this implementation is restricted to a specific instrument and requires a commercial license. This means that other types of widely available laboratory micro-CT instruments cannot have access to *GrainMapper3D* and cannot be used for LabDCT experiments.

To truly boost the use of grain mapping by LabDCT, robust and computationally efficient grain reconstruction methods have been developed, based on forward and back calculations running on a graphical processing unit (GPU) (Fang *et al.*, 2022 ▸ *b*). A first experimental demonstration was reported by Fang *et al.* (2022*a* ▸), providing limited technical details on the implementation. To follow up, in this work we show detailed experimental implementation of this LabDCT technique on a conventional tomography instrument using the two most common types of detectors: CCD and flat panel. An AlCu alloy sample was used as a benchmark for testing these two different instrument configurations, from which 3D grain maps were reconstructed using our previously developed method. To verify the LabDCT grain maps, we performed synchrotron DCT measurement on the same sample and used the synchrotron reconstructed grain map as ground truth for comparison. The results show that most grains were successfully mapped, though some small grains failed to be reconstructed because of their poorer detectability. To provide a guideline for setting up optimal acquisition times, we determined contrast-to-noise ratios (CNRs) as a function of grain size and exposure time for the two different detectors and compared the corresponding grain reconstruction results. The detection limit and spatial resolution are quantified for the current implementation of the LabDCT technique. The performance using different detectors is compared and discussed in detail. Finally, an outlook for further developing the LabDCT technique is presented.

Notably, the purpose of this study is not to provide exhaustive sample measurements or to optimize the grain mapping performance in a general sense for different types of tomography instruments as they differ in source, detector and geometry constraints *etc*. Instead, this study shows an example implementation of grain mapping on a conventional tomography setup and presents the typical performance and limits related to the experimental conditions and instrument, which may serve as a guideline for researchers who wish to implement the LabDCT technique on their own X-ray tomography instruments.

## 2. Materials and methods

### 2.1. Sample

An AlCu alloy (8 wt% Cu) cylindrical rod with a diameter of ∼3 mm and a length of ∼6 mm was annealed at 580°C for 1 h, and then slowly cooled in a furnace at an initial cooling rate of ∼3.5°C min^−1^, with the intention of removing lattice strains. During annealing, the sample was in a solid–liquid two-phase region, where the equilibrium fraction of the solid phase was calculated to be about 83.3% using the lever rule based on the Al–Cu phase diagram. During cooling, the Cu-enriched eutectic phase solidified, giving rise to a continuous layer of precipitates (crystallites smaller than a few micrometres), delineating the grain boundaries of the solid matrix phase. Thereby, the grain shapes of the Al matrix phase can be resolved by absorption contrast tomography due to the significant contrast between the Cu-enriched phase and the face-centered-cubic Al.

A wedge-shaped sample (width × thickness × height ≃ 600 × 450 × 1000 µm) was wire-cut from the heat-treated rod. Sample surfaces were subsequently polished by fine-grid SiC papers before LabDCT and synchrotron measurements.

### 2.2. Laboratory tomography setup

A conventional tomography setup, EASYTOM XL nano focus tomography system manufactured by RX Solutions, was used to implement LabDCT for grain mapping. This setup is compatible with two different sources (L10711 nano source and L8121-03 micro source produced by Hamamatsu Photonics; both can be operated in small-, middle- and large-size modes) and different detectors (CCD, flat panel *etc*.); more details are provided by Fang *et al.* (2022*a* ▸). The micro source is located about 15 mm behind the emission window, whereas the nano source sits very close (∼1 mm) to the window. This makes the nano source more suitable for realizing short sample-to-source distances and consequently larger geometric magnification. Thus it can achieve higher spatial resolutions compared with the micro source, despite the fact that the micro source can provide a higher flux. Tests were performed to confirm this, and hence the nano source was selected for the LabDCT experiments. The maximum acceleration voltage for the nano source is 100 kV. Notably, this source can be operated with an identical maximum current (30.9 µA for middle-size source), independent of the acceleration voltage. Spectrum tests have shown that the total photon flux has a weak correlation with source voltage, whereas the X-ray spectra differ and the fraction of high-energy photons increases with the voltage.

The tomography instrument is equipped with an air bearing sample rotation stage (Lab Motion Systems RT100) with a maximum radial error motion of 178 nm measured at 111 mm from the top surface. This value is far smaller than the effective detector pixel size and can therefore be neglected.

Geometric constraints of the instrument may imply compromises in acquisition geometry. For the instrument described in this study, the source-to-detector distance of the CCD detector is confined to the range 60–505 mm, whereas this range is 230–675 mm for the flat panel. The detectors can move along the beam direction as well as in the lateral direction, whereas it cannot move vertically nor can it rotate. Detector offsets and tilts with respect to the X-ray beam need to be known with high precision and are determined via a fitting procedure described in Section 3.

**Figure 1:**
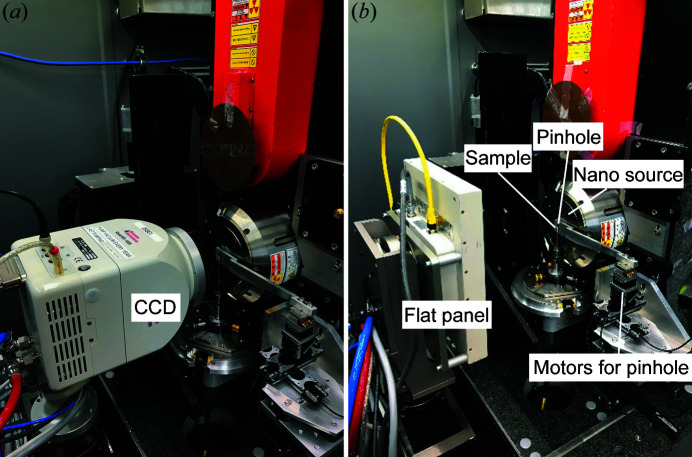
Photographs showing the LabDCT implementation on a conventional tomography setup using a (*a*) CCD (effective width × height = 50.0 × 50.0 mm) and (*b*) flat panel (effective width × height = 184.9 × 233.7 mm). A pinhole is placed between the sample and the nano source, and its position is controlled by three motors. A 2 mm-thick sheet of lead, covering the footprint of the direct beam, is attached onto the detector for DCT acquisition.

For LabDCT measurements, pinholes of different sizes are used to define the dimensions of the illuminated sample volume and the direct beam footprint on the detector, both of which also vary as a function of the distances between these elements. A motorization of the pinhole along the three principal directions with a travel range allowing for complete retraction out of the beam is highly beneficial as it allows for rapid alignment and tuning of the illuminated area in the sample and detector planes, as well as easy switching between conventional imaging and diffraction mode. For the current implementation a series of cylindrical discs made of tungsten (15.9 mm outer diameter and 2 mm thickness) with different sizes of central holes (diameter 100, 200, 400, 1000 and 2000 µm) were prepared. The 400 µm disc, most appropriate for the current sample dimension, was then positioned as close as ∼0.6 mm to the source window with a set of micro-positioning stages (Attocube, Germany). Details of this implementation can be seen in Fig. 1 ▸. A CCD (Quad-RO 4320 produced by Princeton Instruments, 24 µm^2^ pixel size, 2084 × 2084 pixels, coupled to a 150 µm-thick CsI scintillator with a taper 1:1) or a flat panel (PaxScan 2520DX from Varian Medical Systems, 127 µm^2^ pixel size, 1536 × 1920 pixels, using a 600 µm-thick CsI scintillator and an amorphous silicon architecture) was used for recording absorption tomography and DCT projections.

Experimental projections were acquired using *Xact* acquisition software developed by RX Solutions. This software performs image distortion correction and intensity correction but not noise filtering. The maximum exposure time for the CCD detector is 60 s and for the flat panel it is 4 s.

### 2.3. Data collected with the laboratory-based tomography instrument

Measurement on the AlCu alloy sample using the CCD was performed first. Using a conical polychromatic beam emitted from a tungsten target of the nano source (60 kV and 1.8 W, middle size), 384 absorption projections were acquired using an exposure time (*t*_exp_) of 0.5 s for four sample turns, filling in the rotation gaps equally. This resulted in a rotation step of 0.94°. After that, a pinhole with a diameter of 400 µm was placed between the sample and the source, and a beamstop made of Pb with a thickness of ∼2 mm was pasted on paper and placed in front of the detector to attenuate the direct transmitted beam for recording DCT projections. The DCT acquisition was performed with the same source voltage of 60 kV but with a maximum allowed input current of 120 µA on the cathode, reaching a power of 7.2 W and a current of 30.9 µA on the transmission target of the source to maximize the photon flux (note that the current on the target is smaller than the input current on the cathode). Projection images were acquired at a step of 3° over a full sample rotation of 360°. At each rotation angle, six frames were recorded and the exposure time for each frame was 60 s, which is the maximum for the present CCD. Projection images obtained with shorter exposure times are presented in the supporting information.

After the CCD measurement, we manually replaced the detector by the flat panel without changing the sample position. The experimental procedure was repeated: first with an absorption tomography scan (four turns with 800 projections at a rotation step of 0.45° and a source of 60 kV and 1.8 W) and then a DCT scan with 60 kV and 7.2 W source power. The DCT scan was also performed with a rotation step of 3° for a 360° sample rotation; but this time, for each rotation angle, 90 frames were acquired and the exposure time for each frame was 4 s (maximum for this flat panel), resulting in the same accumulated exposure time as the CCD measurement, *i.e.* 360 s per angle.

The main experimental parameters are summarized in Table 1 ▸. The sample-to-source distance (*L*_ss_) was chosen to be very close (∼9.2 mm) for both measurements, while the sample-to-detector distance (*L*_sd_) was selected according to a combination of considerations related to (i) coverage of diffraction angles, (ii) effective pixel size in the sample plane and (iii) size of the direct beam footprint (to be covered by the beamstop). Note that further reduction of the sample-to-source distance (*i.e.* increasing the opening angle of the cone beam using a larger pinhole) would be beneficial for optimizing flux density but also increases the footprint of the direct beam and therefore reduces the effective detector region for recording the diffraction signals. Ultimately, the effective pixel size in the sample plane for the flat panel is slightly bigger than that for the CCD. The source voltage was selected by considering the balance between a proper transmission of the X-ray beam and a suitable coverage of the most probable diffraction spot energies. In the case of the AlCu alloy sample studied here, diffraction spots from the first four {*hkl*} families mainly have photon energies in the range 15–45 keV according to our forward simulation (Fang *et al.*, 2020 ▸) under the current experimental conditions. With additional LabDCT tests, a source voltage of 60 kV was chosen. More detailed testing results for key experimental parameters, including *L*_sd_, source voltage and source size, are presented in the supporting information.

An absorption tomographic volume with a voxel size of 2.7 µm was reconstructed from the absorption projections, using the *Xact* reconstruction software developed by RX Solutions. In the sample volume, three phases were identified: Al matrix, Cu-enriched eutectic phase and cavities. A sample volume mask was defined by segmenting the Al matrix phase. Since there are many fewer tomography projections recorded by the CCD (384 projections) than the flat panel (800 projections), the reconstructed tomography volume from the CCD acquisition has a poorer quality than that from the flat panel. As a result, the segmented volume mask from the CCD is noisier than that from the flat panel, and the Cu-enriched eutectic phase is less well resolved.

The sample volume mask, together with the DCT projection images and acquisition geometry parameters, were used as input for 3D grain reconstruction using the method reported in our previous work (Fang *et al.*, 2022 ▸ *b*). A number of the reconstructions were performed using only sub-samples of the available projection data in order to mimic shorter exposure times. Currently, running the grain reconstruction requires a MATLAB license, but the programming code could be translated to other open source programming languages (*e.g.* Python). Details about the grain reconstruction procedure are presented in Section 3.

### 2.4. Synchrotron diffraction contrast tomography

Synchrotron DCT measurements were performed on beamline ID11 at the European Synchrotron Radiation Facility (ESRF). The same AlCu alloy sample was illuminated by a parallel monochromatic beam with an energy of 43.64 keV. An sCMOS (Andor Marana) detector with 2048 × 2048 pixels was placed at a distance of 7.2 mm from the vertical rotation axis. Diffraction signals were recorded by the outer area of the detector, while the transmitted direct beam was attenuated by a beamstop and recorded by the central area of the detector. The detector was coupled to a 10 µm-thick transparent luminescent screen via a 7.5× objective lens, resulting in an effective pixel size of 1.6 µm. A series of 3600 equally spaced projections over 360° sample rotation were acquired with an exposure time of 0.15 s for each projection. A 3D grain map, together with tomographic volume with a voxel size of 1.6 µm, was reconstructed using the method described by Ludwig *et al.* (2009 ▸) and Reischig *et al.* (2013 ▸).

### 2.5. Comparison of reconstructed grain maps from LabDCT with synchrotron DCT

Owing to its superior detection limit and spatial resolution (Reischig *et al.*, 2013 ▸; Renversade *et al.*, 2016 ▸; Fang *et al.*, 2021*b* ▸; McDonald *et al.*, 2021 ▸), the grain map reconstructed from the synchrotron DCT was considered as ground truth and the result is referred to as SR-DCT. To compare the orientation and spatial deviation between the LabDCT grain maps and SR-DCT, the SR-DCT dataset was registered to the grain volume of the LabDCT dataset by resampling (voxel size increased from 1.6 to 2.7 µm, being the same as that used in the LabDCT datasets), rotating and translating the SR-DCT volume, using the method reported by Fang *et al.* (2021*b* ▸). As the grain shapes are also revealed by the grain boundary precipitation of the Cu-enriched phase, tomography volumes, reconstructed by the synchrotron and laboratory measurements, respectively, were used to further verify the accuracy of the volume registration as well as to check the accuracy of the grain shape reconstruction.

To assess how effective the grain indexing is, grains were paired between the LabDCT and SR-DCT datasets on the basis of their orientations and spatial locations. All the grains are classified into three categories: (1) true positively indexed grains (TPs), including one-to-one indexed and one-to-multi indexed ones (a grain in the SR-DCT dataset reconstructed as multiple grains with similar orientations in the LabDCT dataset); (2) false negatively indexed grains (FNs), which exist in SR-DCT but are not found in the LabDCT dataset; (3) false positively indexed grains (FPs), which are indexed in the LabDCT dataset but not in SR-DCT. To evaluate the indexing accuracy, the *F*_1_ score was calculated from the precision (*P*) and sensitivity (*S*), *i.e. F*_1_ score = 2*P* *S*/(*P* + *S*), where *P* = TP/(TP + FP) and *S* = TP/(TP + FN). The *F*_1_ score has a value between 0 and 1, with a value closer to 1 corresponding to a better indexing performance. Note that the *F*_1_ score is a metric to evaluate the overall indexing performance and is calculated on a grain-by-grain basis, as reported elsewhere (Fang *et al.*, 2021*a* ▸,*b* ▸).

For the paired grains, disorientations (Δ_OR_) were calculated by the *MTEX* toolbox (Bachmann *et al.*, 2010 ▸) to evaluate the orientation resolution. To evaluate how good the reconstruction is for the grain shape (considered to be the spatial resolution), the deviations in the grain center of mass (Δ_grain_) and grain boundary deviation (δ_GB_) for each grain pair were computed. δ_GB_ was calculated as

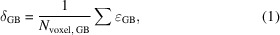

where ɛ_GB_ is the Euclidean distance between a grain boundary voxel in the SR-DCT dataset and the nearest voxel on the boundary of the paired grain in the LabDCT dataset; *N*_voxel, GB_ is the total number of grain boundary voxels in the SR-DCT dataset. More details about the method for the comparison are given by Fang *et al.* (2021*a* ▸,*b* ▸).

## 3. Procedure of grain reconstruction for LabDCT

Let us define a right-handed laboratory coordinate system with the beam direction *x*, horizontal direction *y* and vertical (parallel to rotation axis) direction *z*. To reconstruct a 3D grain map, five inputs must be prepared: (1) spot segmented images processed from the diffraction projections; (2) volume mask determined from the segmentation of the tomography volume (see Section 2.2); (3) geometry parameters including *L*_ss_, *L*_sd_, source offsets in the horizontal and vertical directions (denoted *S_y_* and *S_z_*, respectively), detector offsets horizontally (det*y*0) and vertically (det*z*0), and tilts about the *x*, *y* and *z* axes (*φ_x_*, *φ_y_* and *φ_z_*, respectively); (4) lattice parameters of the sample; (5) reconstruction parameters. In the current work, we restrict our LabDCT grain mapping to samples with *a priori* known crystal structures and negligible lattice strains.

**Figure 2:**
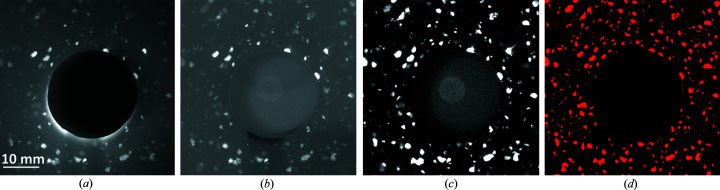
Processing of LabDCT images obtained from CCD measurements. (*a*) Experimental image averaged by six frames, (*b*) flat-field corrected image, (*c*) image after rolling median subtraction, and (*d*) spot segmented image where the spots are shown in red with a pixel value of 1 and the rest is shown in black with a pixel value of 0.

We choose the LabDCT measurement on the AlCu alloy sample with the CCD detector to illustrate the grain reconstruction procedure. Fig. 2 ▸ shows the image-processing procedure. Fig. 2 ▸(*a*) shows one experimental image averaged over six CCD frames, corresponding to an exposure time of 360 s (6 frames × 60 s per frame). Then, a flat-field correction using the same method as reported by Lindkvist *et al.* (2021 ▸) was applied, and the contrast between the spot and the background is enhanced [Fig. 2 ▸(*b*)]. Subsequently, a rolling median background correction was performed [Fig. 2 ▸(*c*)]. Last, a Laplacian of the Gaussian based method (Lind, 2013 ▸; Bachmann *et al.*, 2019 ▸; Fang *et al.*, 2021*b* ▸) was used to segment the diffraction spots [Fig. 2 ▸(*d*)].

Grain reconstruction was performed using the method reported in our previous work (Fang *et al.*, 2022 ▸ *b*). The grain reconstruction algorithm mainly comprises two steps: (1) indexing a seeding voxel *i* by maximizing its completeness (*C*_seed *i*_), defined as the number of intersected spots between forward calculation and experiment divided by the number of forward calculated spots, to derive its orientation; (2) growing a region by assigning the indexed seeding orientation to neighboring voxels that fulfill growth criteria. For a voxel *j* around the seeding voxel *i*, it will only be accepted into the grown region when its completeness (*C*_voxel *j*_) stays above a certain percentage of *C*_seed *i*_ and its new median distance (*D*_median_) is not larger than its old value. In this work, the reconstruction parameters (given in Table 2 ▸) were kept the same for all grain reconstructions. To reconstruct grain maps for the Al matrix, a lattice parameter of 4.0498 Å with a face-centered-cubic structure and the first four {*hkl*} families (*i.e.* {111}, {002}, {022} and {113}) were used. Seeding voxels were generated iteratively with an increasing ‘sample gridding level’, from level 1 (coarse sampling with a minimum distance of 45 pixels between seeding voxels) to 11 (fine sampling with a minimum distance of 3 pixels between seeding voxels). This results in a total of ∼11 000 seeding voxels for testing. All the grain reconstructions were performed with an NVIDIA Tesla V100-PCIE-32GB GPU running in the MATLAB software on the ESRF computing cluster. The reconstruction time varied between 12 and 48 h.

Notably, among the reconstruction parameters the minimum completeness (*C*_min_) has the most critical effect on the number of correctly reconstructed grains. Grain reconstructions with *C*_min_ values of 0.4, 0.35, 0.30 and 0.25 were performed to test the choice for this sample. The results show that the grain reconstruction with *C*_min_ = 0.30 gives the highest number of TPs, while no FPs are present. However, the FPs start to appear in the grain reconstruction with *C*_min_ = 0.25. Therefore, *C*_min_ = 0.30 was set for all the reconstructions in this work.

**Figure 3:**
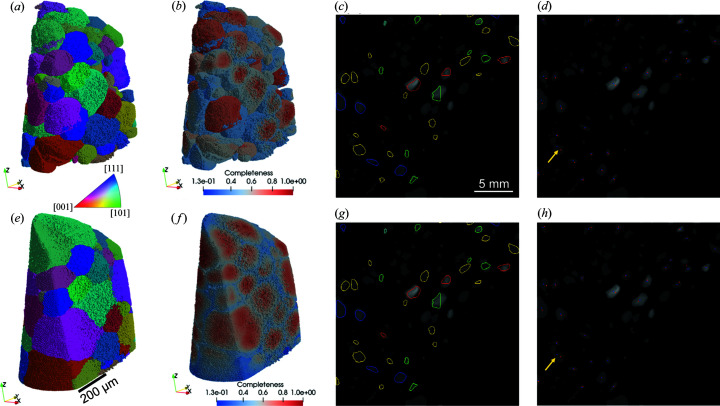
Grain reconstructions before (upper row) and after (bottom row) geometry fitting. (*a*) and (*e*) 3D grain maps colored by IPF-*z*, (*b*) and (*f*) 3D completeness map, (*c*) and (*g*) outlines of forward spots overlaid onto the spot segmented images, and (*d*) and (*h*) centers (intensity weighted) of forward (marked by red dots) and experimental (marked by blue dots) spots overlaid onto the spot segmented image.

Fig. 3 ▸ shows grain reconstruction results from the CCD measurement. Note that the grains are colored in the *z* direction of the inverse pole figure (IPF-*z*) throughout the whole paper. Fig. 3 ▸(*a*) shows a first grain map, reconstructed using the raw geometry, and the corresponding completeness map is shown in Fig. 3 ▸(*b*). Since the goal of this initial grain map was only to fit the geometry, the reconstruction was interrupted when ∼80% of the sample volume was reconstructed. To fit the geometry, relatively large grains with relatively high completeness were selected (22 grains in this case) to perform forward simulations, from which the forward spots are overlaid onto the spot segmented image [Fig. 3 ▸(*c*)]. Distances between the forward and experimental spots were calculated [Fig. 3 ▸(*d*)] and the fitting was subsequently carried out to minimize the average spot distance 〈*D*_spot_〉, resulting in improvements in both the overlay of the forward spots onto the experimental ones [Fig. 3 ▸(*g*)] and the spot center distances [Fig. 3 ▸(*h*)]. Notably, experimental spots may be overlapped as marked by the yellow arrows in Figs. 3 ▸(*d*) and 3 ▸(*h*). This will lead to inaccurate determination of the spot centers, and thus may influence the fitting results and give an overestimation of 〈*D*_spot_〉. Although we tried to exclude the overlapped spots from the fitting by setting up thresholds of spot distances and size differences, we cannot completely rule out the overlapped ones. Therefore, the derived 〈*D*_spot_〉 should be considered as overestimated. Table 3 ▸ summarizes the geometry parameters before and after the fittings for both CCD and flat panel measurements.

Fig. 3 ▸(*e*) shows the final grain reconstruction obtained with the fitted geometry. Fig. 3 ▸(*f*) shows the corresponding completeness map. Compared with Fig. 3 ▸(*b*), an overall increase of the completeness can be seen in Fig. 3 ▸(*f*) and the completeness gradient from grain central regions towards the grain surfaces is more visible. Note that in Fig. 3 ▸(*f*) the grains located in the bottom edge region have relatively small completeness values even though they are large. This is because they suffered a partially illuminated situation, *i.e.* not always in the field of view at all rotation angles during the measurement because of the circular pinhole shape.

## 4. Results

### 4.1. Comparison of grain maps between LabDCT and synchrotron DCT

Grain maps reconstructed from the flat panel and CCD measurements with the longest exposure time (*i.e.* 360 s), referred to as Lab-flat-panel and Lab-CCD, respectively, are compared with the registered grain map of the synchrotron DCT measurement (considered as ground truth).

Table 4 ▸ provides a summary for the indexing comparison. In both Lab-flat-panel and Lab-CCD, no FPs are found, indicating a very high indexing precision. Lab-CCD has the same number of one-to-one indexed grains as Lab-flat-panel. However, Lab-CCD has a few more one-to-multi indexed grains, for which a relatively large grain in SR-DCT typically pairs with a large grain together with a tiny grain apart in the LabDCT grain map in this case. The average disorientations for the TPs are Δ_OR_ = 0.08 ± 0.08° for both LabDCT datasets, with the majority of them being smaller than 0.05°. The grain centroid deviations are Δ_grain_ = 2.1 ± 1.2 pixels for Lab-flat-panel and Δ_grain_ = 2.4 ± 1.1 pixels for Lab-CCD (here 1 pixel = 2.7 µm for the sample). The *F*_1_ score values obtained are very close between the two LabDCT datasets (see Table 4 ▸). Notably, if we exclude the FNs on the sample surface, the *F*_1_ score will be 1 for both datasets.

**Figure 4:**
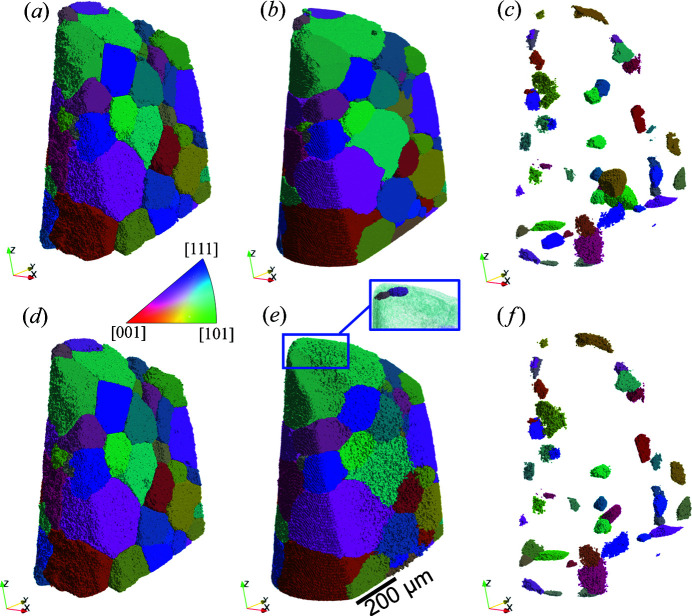
Comparison of grain maps between synchrotron and the two laboratory-based DCT datasets obtained from measurements with the flat panel (upper row) and CCD (bottom row), respectively. (*a*) and (*d*) SR-DCT grains that are correctly indexed in the corresponding LabDCT dataset, (*b*) and (*e*) correctly indexed grains in the LabDCT dataset (TPs), and (*c*) and (*f*) SR-DCT grains that are not indexed in the corresponding LabDCT dataset (FNs). An inset in (*e*) makes the big green grain semi-transparent to visualize the locations of the two grains colored in light and dark pink at the top.

Fig. 4 ▸ shows a visualization of the TPs and FNs for the comparison between the two LabDCT datasets and SR-DCT. Compared with the TPs in SR-DCT [Figs. 4 ▸(*a*) and 4(*d*)], most of the grains have been correctly indexed in both Lab-flat-panel [Fig. 4 ▸(*b*)] and Lab-CCD [Fig. 4 ▸(*e*)] and their shapes are close to those found by SR-DCT, though with some visible differences, which are not exclusively linked to the location of FNs. All the FNs lie on the sample surface, and they are relatively small and are mostly the same for reconstructions obtained from the CCD and flat panel detectors [Figs. 4 ▸(*c*) and 4(*f*)].

**Figure 5:**
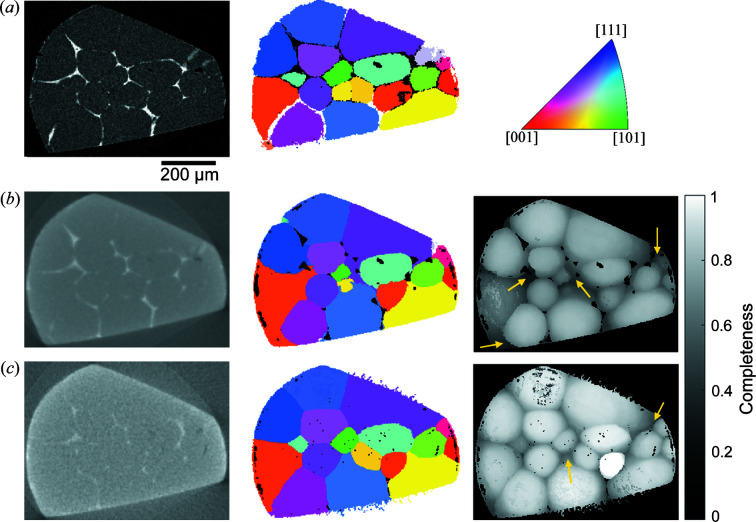
Comparison of 2D sections from tomography volumes and grain maps shown in the left and middle columns, respectively, and the corresponding completeness map shown in the right column. (*a*) Synchrotron, (*b*) Lab-flat-panel and (*c*) Lab-CCD.

A closer comparison is shown in Fig. 5 ▸ on a selected 2D slice. Here, tomographic slices (left column in Fig. 5 ▸) are also shown with an intention to demonstrate that (i) grain contours obtained from SR-DCT are consistent with locations of the grain boundary precipitates [visible as bright contrast in Fig. 5 ▸(*a*)] and (ii) the volume registration is accurate. Comparing the 2D grain slices (middle column), Lab-CCD is more similar to SR-DCT than Lab-flat-panel because of the better grain shapes and more grains appearing in this slice. Differences in small grain regions are visible in both LabDCT grain maps and examples are marked by the yellow arrows in the completeness maps (right column), showing relatively low completeness values. We tried to use the correct grain orientations from SR-DCT to compute the completeness for these regions. The resulting completeness is found to be even smaller than the current values. This suggests that imperfect grain shapes are caused by insufficient diffraction signals rather than the reconstruction method.

**Figure 6:**
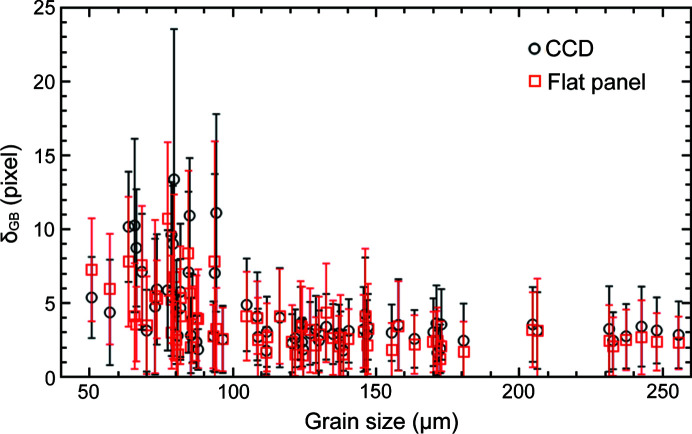
Grain boundary deviation (δ_GB_) as a function of grain size for Lab-CCD and Lab-flat-panel. Error bars correspond to the standard deviations of δ_GB_. Here 1 pixel corresponds to 2.7 µm.

Fig. 6 ▸ shows grain boundary deviation (δ_GB_) as a function of grain size for 69 commonly indexed grains to quantify the accuracy of the reconstructed grain shapes with respect to SR-DCT. The figure shows that δ_GB_ behaves similarly in the two LabDCT datasets, remaining at a constant of 2–3 pixels (δ_GB_ = 2.97 ± 2.30 pixels for Lab-CCD and δ_GB_ = 2.64 ± 2.38 pixels for Lab-flat-panel with 1 pixel = 2.7 µm) when the grain size is >100 µm, below which δ_GB_ starts to increase. Similar behavior has also been observed by Fang *et al.* (2021*a* ▸,*b* ▸) and is considered as a general characteristic for the LabDCT technique. The reason for increasing δ_GB_ with decreasing grain size below a certain size is mainly the poorer diffraction signals, resulting in worse spot segmentation and fewer spots to be successfully segmented. These further lead to larger errors in the determination of the spot centers and shapes, thereby influencing the grain boundary accuracy.

Another consequence of the poorer signals is that large neighboring grains may have higher completeness and ‘grow’ into the regions which should have been occupied by the small missing grains. This has been observed as marked by yellow arrows in Fig. 5 ▸.

### 4.2. Contrast-to-noise ratio as a function of exposure time

**Figure 7:**
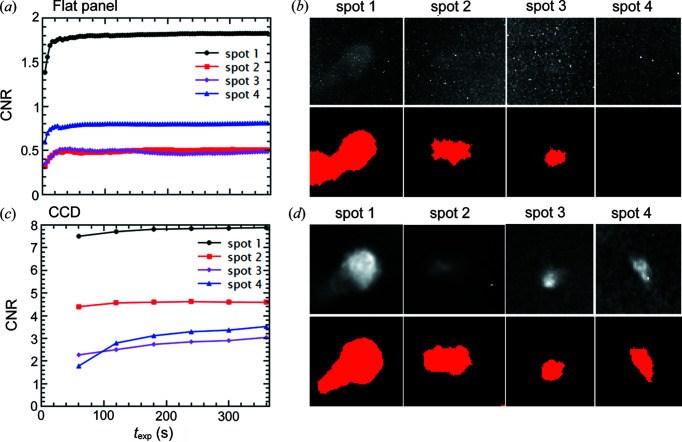
CNR as a function of exposure time for four spots tracked in both (*a*) flat panel and (*c*) CCD measurements. The spot cropped images obtained from *t*_exp_ = 360 s (upper row) and the corresponding spot segmented images (lower row) are shown in (*b*) for flat panel and (*d*) for CCD measurements. Spot 1 corresponds to grain 25, 1 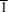 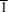 , rotation angle ω = 288°, average spot energy *E*_spot_ = 18.2 keV; Spot 2: grain 79, 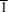 1 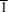 , ω = 288°, *E*_spot_ = 14.3 keV; Spot 3: grain 111, 1 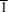 1, ω = 273°, *E*_spot_ = 16.3 keV; Spot 4: grain 142, 1 1 1, ω = 216°, *E*_spot_ = 18.9 keV. The sizes of grains 25, 79, 111 and 142 are 163.4, 85.3, 57.0 and 36.4 µm, respectively. Grains 25, 79 and 111 are all indexed in the LabDCT datasets, whereas grain 142 is not, in either one.

The CNR is determined for individual spots in raw frames before any image processing, and the calculation method is illustrated in Appendix *A* [App appa]. Fig. 7 ▸ shows CNR as a function of exposure time (*t*_exp_) for four {111} spots from grains with different size levels. It can be seen in Fig. 7 ▸(*a*) that CNR saturates in ∼20 s for all four spots from the flat panel measurements, whereas CNR saturates at different times for the CCD data as shown in Fig. 7 ▸(*c*). Comparing the CNR values in Figs. 7 ▸(*a*) and 7 ▸(*c*), the values from the CCD measurement are significantly higher than those from the flat panel measurement. Although the CNR values are rather low, as plotted in Fig. 7 ▸(*a*), the spots can still be identified and segmented except for spot 4 (from a small grain) as shown in Fig. 7 ▸(*b*). Given higher CNR values, the spot segmentation looks more accurate for the CCD measurement [Fig. 7 ▸(*d*)].

Interestingly, whether or not a spot can be successfully segmented is not linearly proportional to its CNR value, as shown by spot 4 in the flat panel measurement; it is not segmented but it has a higher CNR value (∼0.8 at *t*_exp_ = 360 s) than spots 2 and 3 (∼0.5 at *t*_exp_ = 360 s), both of which are segmented. The reason is that the final spot segmentation not only depends on the CNR in the raw image but also depends on the subsequent processing (flat-field correction, rolling median and filtering *etc*.). This means the spot segmentation may also be influenced by local spot regions as well as global background intensity. By tracking additional spots, it is found that spots with CNR < 1 have a high chance of not being segmented and CNR values vary dramatically even for the same grain, depending on spot energy, *hkl* reflection, rotation angle ω and the spot location on the detector (corners have higher background noise than the inner region).

**Figure 8:**
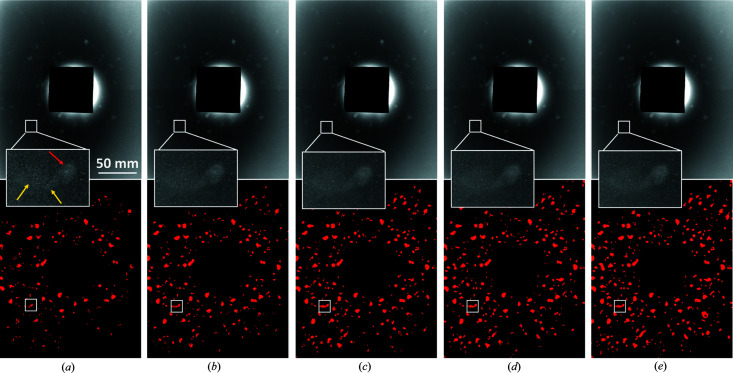
Raw LabDCT images acquired by the flat panel (upper row) and the corresponding spot segmented images (bottom row) at ω = 288°. Images are averaged by 1, 5, 15, 30 and 90 frames in (*a*)–(*e*), corresponding to an exposure time of 4, 20, 60, 120 and 360 s, respectively. Zoom-ins show the spot 1 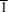 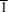 from grain 25 [marked by the red arrow in (*a*); see its CNR in Fig. 7 ▸] and white boxes mark the corresponding segmentation in the bottom images. Note that the spot of interest partially overlaps with two other spots [marked by the yellow arrows in (*a*)], and hence they are segmented as one single spot.

Fig. 8 ▸ shows raw LabDCT images from the flat panel measurement and the corresponding spot segmented images for *t*_exp_ = 4, 20, 60, 120 and 360 s. It can be clearly seen that the number of segmented spots increases with *t*_exp_ [the number of spots is 190, 240, 268, 292 and 343 in Figs. 8 ▸(*a*)–8 ▸(*e*), respectively]. Zoom-ins of one large spot (that is, spot 1 in Fig. 7 ▸) show that its background noise is significantly reduced from *t*_exp_ = 4 to 20 s; then it does not change too much, as can also be seen from the segmented shapes.

### 4.3. Grain reconstruction as a function of exposure time

**Figure 9:**
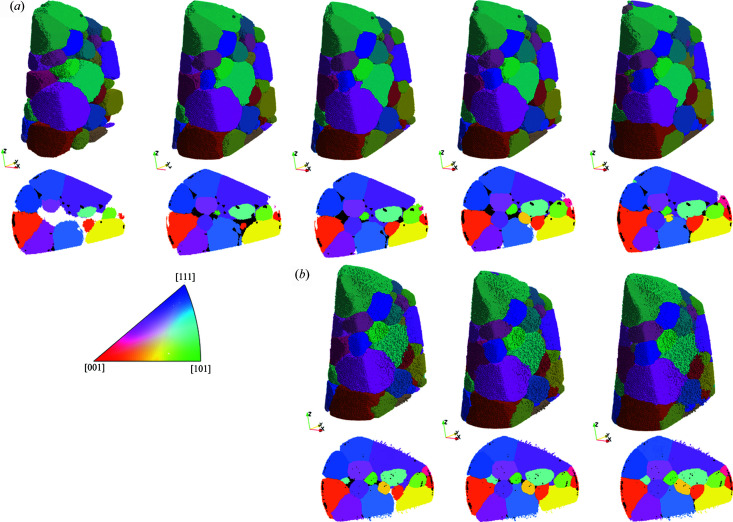
Comparison of grain reconstructions with different exposure times for (*a*) flat panel (left to right: *t*_exp_ = 4, 20, 60, 120 and 360 s) and (*b*) CCD measurements (left to right: *t*_exp_ = 60, 120 and 360 s).

Grain maps are reconstructed from LabDCT images with different exposure times for both flat panel and CCD detectors. Fig. 9 ▸ shows a comparative view of the grain maps. It can be seen in Fig. 9 ▸(*a*) that grain volume increases with *t*_exp_, with an increasing number of relatively small grains being reconstructed. The grain shapes also improve, increasingly conforming to the empty space with increasing *t*_exp_. Notably, the majority of the grains are reconstructed even with *t*_exp_ = 4 s for the flat panel detector. When *t*_exp_ increases to 20 s, the sample volume is nearly filled, and this filling ratio continues to improve with longer exposure time. For the CCD measurement, the shortest resolved exposure time in this work is 60 s. It can be seen in Fig. 9 ▸(*b*) that the grain map quality with *t*_exp_ = 60 s is already quite close to the best one (*t*_exp_ = 360 s). Comparing Figs. 9 ▸(*a*) and 9 ▸(*b*), a similar relationship of grain map quality is observed as a function of *t*_exp_ when its value is ≥60 s between the flat panel and CCD reconstructions.

A quantitative indexing comparison is summarized in Table 5 ▸ to show that an increasing number of grains are correctly indexed with increasing *t*_exp_ for both detectors. A total of 88% of the grain volume is reconstructed with *t*_exp_ = 4 s for the flat panel, and 98% is reconstructed when *t*_exp_ = 20 s for the flat panel and 60 s for the CCD.

## 5. Discussions

### 5.1. Detection limit and spatial resolution of the current LabDCT

**Figure 10:**
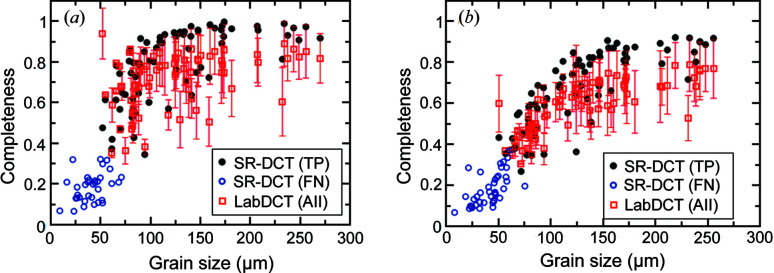
Completeness as a function of grain size for measurements of (*a*) CCD and (*b*) flat panel detectors. Completeness values are plotted as an average and an error bar for all the LabDCT grains, while only the grain centroid completeness is plotted for SR-DCT grains. These grains are classified into TPs and FNs.

It can be seen from Figs. 4 ▸ and 5 ▸ and Table 4 ▸ that the current LabDCT implementation on the AlCu sample is capable of resolving all the relatively large grains, but has a detection limit of ∼50 µm, below which the reconstruction fails. To explain this, the completeness is plotted as a function of grain size using grain maps from both LabDCT and SR-DCT as input. Fig. 10 ▸(*a*) shows the completeness values for all the reconstructed grains from Lab-CCD, together with the values for grain centroids of the TPs and FNs in SR-DCT. The figure shows that most of the FNs have completeness values smaller than 0.3, which is the minimum completeness for the reconstruction. Three FNs have completeness values slightly above 0.3, which would be expected to be ideally reconstructed. However, the reason for not being able to reconstruct these grains is that the completeness values are higher with other grain orientations than using orientations of these grains. This indicates that the grain reconstruction algorithm does not hinder the improvement of the detection limit; it is the detectability of the diffraction signals controlling the current detection limit. A similar behavior is seen in Fig. 10 ▸(*b*) for the flat panel, where the completeness values for most of the FNs fall below 0.3 and the other five have values above. The latter has the same reason as for the CCD data. Notably, in both Figs. 10 ▸(*a*) and 10 ▸(*b*), one TP with a grain size of 50.7 µm has a much higher completeness from both LabDCT datasets than from SR-DCT. This is found to be due to a significantly different orientation (a disorientation of ∼0.4°). On closer inspection of the SR-DCT dataset, the corresponding grain shows a distinct sub-grain structure, and one of these subgrains has been indexed in SR-DCT as an additional small grain with a misorientation of 0.45°.

The spatial resolution, expressed by the grain boundary deviation [similar to Fang *et al.* (2021 ▸ *a*, ▸ *b*)], is at a constant level of ∼2–3 pixels (∼5–8 µm), showing a reasonably good performance. However, the spatial resolution becomes worse as the grain size becomes smaller than 100 µm (see Fig. 6 ▸). This is related to the number of spots for reconstruction. As can be seen in Fig. 10 ▸, the completeness values also start to decrease from a relatively constant value with decreasing grain size below than 100 µm, corresponding to fewer spots detected/segmented for reconstruction.

### 5.2. Characteristics of the different detectors

The grain maps reconstructed from the flat panel and the CCD measurement data are somewhat comparable, although the CCD gives a marginal improvement in the grain indexing (see the *F*_1_ score in Table 4 ▸). However, given the CNR for the CCD is much higher than that for the flat panel (see Fig. 7 ▸), one would expect that the performance of both the detection limit and spatial resolution for the CCD must be significantly better than for the flat panel. There are two main reasons: (1) spot segmentation is not linearly proportional to CNR as explained in Section 4.2; (2) the flat panel covers a larger range of scattering angles and thus records more spots than the CCD. Assuming a diffraction event occurs at the origin, the flat panel measurement covers a scattering angle (2θ) range from 7.4 to 33.6°, while 2θ is in the range 12.5–32.0° for the CCD measurement. Although the maximum accessible angle (2θ_max_) is only slightly larger, the flat panel has a significantly smaller 2θ_min_, which is beneficial for recording spots from lower-order {*hkl*} reflections (usually brighter spots). This suggests that a detector with a larger recording area can be preferable in the presence of geometrical constraints (minimum distances of *L*_ss_ and *L*_sd_) limiting the accessible 2θ range.

**Figure 11:**
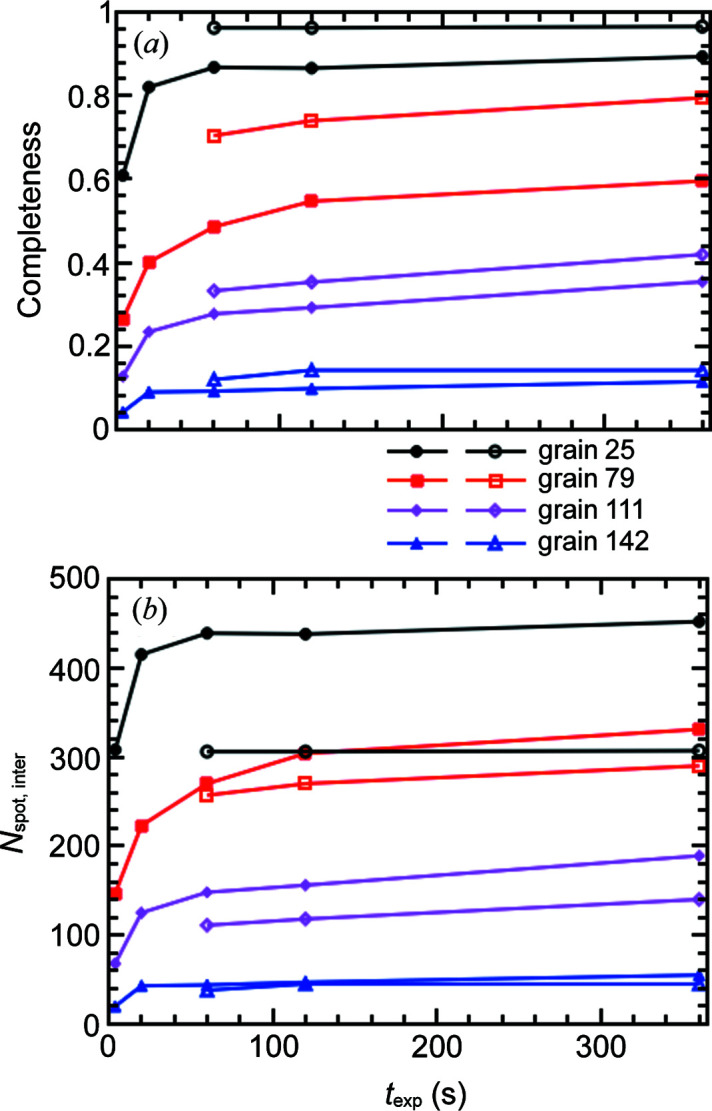
(*a*) Completeness and (*b*) number of intersected spots (*N*_spot, inter_) between forward simulation and experiment as a function of exposure time for four selected grains, whose CNRs are plotted in Fig. 7 ▸. Closed symbols correspond to the flat panel data, while the open symbols correspond to the CCD data.

As shown in Fig. 11 ▸(*a*), the completeness values for all four selected grains, regardless of whether they are indexed or not in the LabDCT datasets, are always lower in the flat panel measurements than the CCD measurements for the same exposure time. This is consistent with the plots of CNR (see Fig. 7 ▸). However, Fig. 11 ▸(*b*) shows that the number of intersected spots is actually higher in the flat panel data than in the CCD data. This means that more spots were used for reconstructing the grain shapes and partly explains why the spatial resolution of the flat panel measurement is very close to the CCD measurement. Fig. 11 ▸ also explains the observation in Fig. 9 ▸ that the grain maps are continuously improving with increasing exposure time, although it appears that the CNR quickly saturates for some individual spots (*e.g.* within 20 s for the flat panel).

It is found that the two detectors have different advantages and disadvantages. Compared with the CCD, the flat panel has lower sensitivity to the diffraction signals and has a larger pixel size, thus requiring a longer *L*_sd_ to achieve comparable effective pixel size. However, the flat panel is much cheaper and easier to implement (*e.g.* no cooling is required) and has a faster readout time. Since our grain reconstruction method is working on spot binarized images rather than on spot intensities, the poorer sensitivity of the flat panel does not cause too much harm in the final grain map quality; as demonstrated with the present AlCu alloy sample, the quality is comparable to the CCD result. Nevertheless, the CCD has a better sensitivity and gives better CNRs, making it more advantageous in increasing the completeness value for a given grain size.

### 5.3. Geometry optimization

Although geometry optimization for the LabDCT experiments presented in this work has been mostly concentrated on the sample-to-detector distance (see the supporting information), considerations on the sample-to-source distance, the position/choice of the aperture to confine the incident beam and the beamstop to cover the footprint of the direct beam are also important to optimize the LabDCT geometry. For the basic acquisition protocol described here (*i.e.* single rotational scan of a fully illuminated sample cross section), the dimensions of the sample may have to be adapted to the grain size of the material so as to limit the through-thickness dimension to about ten grain diameters in order to limit diffraction spot overlaps on the detector. Shorter source-to-sample distances will increase the photon flux at the sample position, but also lead to a wider opening angle of the cone beam and hence reduced area for the collection of diffraction signals on the detector – a compromise has to be found here. The sample and the aperture should be placed close to the source for increasing the photon flux at the sample position. Placing the aperture close to the sample allows us to reduce scattering and fluorescence from the aperture itself, thereby reducing the high-background area close to the direct beam and increasing the effective area for detecting diffraction spots on the detector. Notably, the use of a circular aperture as in the current implementation is not optimal, because it can lead to partial illumination of sample sub-volumes as described in Section 3. A selection of rectangular windows with variable aspect ratios would provide better flexibility to adapt to samples of different dimensions and grain size.

The choices of the sample-to-detector distance and the beamstop should take into account a combination of the diffraction angle coverage, effective pixel size in the sample plane, footprint size of the direct beam, spot CNR and probability of spot overlap. These parameters altogether may not be straightforward to be sort out for an optimal geometry setting, whilst they strongly depend on sample characteristics and scientific questions associated with the sample. In practice, tests of diffraction projections with different combinations of choices may be useful to select a geometry setting to perform decent LabDCT experiments for a given sample.

### 5.4. Outlook for future development of the LabDCT technique

It is demonstrated that ‘fast’ grain mapping by LabDCT is possible. As shown in Fig. 9 ▸ and Table 5 ▸, 88% of the grain volume is reconstructed with an exposure time of 4 s. This corresponds to a dramatic decrease in total scanning time from typically ∼12 h to only ∼10 min for 121 LabDCT projections. Even with the exposure time increasing to 20 s, the total scanning time will be less than 1 h, while the grain map quality will be significantly improved (*e.g.* 98% of the grain volume was reconstructed). This much shorter scanning time will make LabDCT measurements compatible with the scanning time of conventional tomography scans using laboratory-based X-rays. Therefore, it opens the possibility to perform time-lapse LabDCT observations of processes like grain growth on conventional X-ray instruments.

Although the spatial resolution is rather close, the detection limit of the current LabDCT implementation on the conventional tomography setup is inferior to the commercial one [*e.g.* Zeiss Xradia 520 Versa (Fang *et al.*, 2021 ▸ *b*; McDonald *et al.*, 2021 ▸)]. This is mainly due to the usage of different sources and detector systems. A measurement on the spectrum of the X-ray source for the current instrument shows that the spectrum has a peak intensity around 10 keV (related to the *L*α edge of tungsten anode material) and is not yet optimal for maximizing the performance of the LabDCT technique. Although different materials favor high fluxes at different energy ranges, it is preferable to have continuous high fluxes in the energy range 15–80 keV for measuring typical metallic materials.

Improving detective quantum efficiency and reducing background noise is also an effective approach to improving the detection limit for LabDCT. New direct photon counting detectors show promising results in X-ray imaging and tomography (*e.g.* Bellazzini *et al.*, 2015 ▸; Ballabriga *et al.*, 2020 ▸). With these detectors, ideally, we expect to have sharp images with very low background noise and very small point spread, and even have a capability to resolve X-ray energies with the ‘color’ detectors. In practice, we also tested a prototype CdTe direct photon counting detector for LabDCT grain mapping. The recorded diffraction images were sharp and have a smaller point spread compared with both the flat panel and the CCD. However, the dimension of this CdTe detector, as well as some other issues related to the use of this detector, mean that the final performance is not yet optimal. Nevertheless, we anticipate that this new generation of detectors will lead to significant improvements of the LabDCT technique.

There is room to further develop the reconstruction algorithm to improve the detection limit. Currently, a single value for the minimum completeness is used to determine the acceptance or rejection of an orientation indexing. In general, this value should not be so high as to miss TPs, and also should not be so low as to induce the reconstruction of FPs. As shown in Fig. 10 ▸, small grains inevitably have low completeness values, making them more susceptible to unsuccessful indexing. This means that the smallest reconstructed grain in the final grain map would be generally larger than the size inferred from the smallest spot detected on the detector, because its maximum completeness value is more likely to be smaller than the minimum completeness value threshold. One possible way to overcome this limit is to perform successive reconstructions with decreasing minimum completeness parameter values. At each iteration the spots that can be reliably paired with the already-indexed grains will be removed. In this way, rather low minimum completeness values can be used to favor the reconstruction of the small grains and simultaneously reduce the probability of reconstructing FPs. Ultimately, a smaller detection limit could be achieved.

The current reconstruction algorithm works on spot binarized images, *i.e.* independent of spot intensities. This is very efficient for reconstructing grain maps, and beneficial for measurement data when the CNRs of the diffraction spots are low but still segmentable (*e.g.* the flat panel data in this work). However, working on binarized images also means that intra-granular orientations within individual grains cannot be retrieved. To expand the current LabDCT technique from only strain-free samples to deformed samples, a further development of the reconstruction algorithm taking into account the spot intensities will be required.

## 6. Conclusions

In this study, a successful implementation of grain mapping by LabDCT has been demonstrated on a conventional tomography setup using a flat panel detector and a CCD. The grain reconstruction procedure using our previously developed method is presented in detail. The grain maps obtained from an AlCu alloy sample were compared with the ground truth from a synchrotron measurement. CNRs are studied as a function of exposure time and the resulting grain reconstructions are quantitatively assessed. A comprehensive discussion on the current performance, characteristics of different detectors and the outlook is presented. The main conclusions are listed as follows.

(1) The grain maps from the LabDCT measurements are reasonably well reconstructed with respect to the synchrotron ground truth. Most of the grains are correctly reconstructed with an average orientation accuracy of ∼0.08° and a spatial resolution of ∼2–3 pixels (*i.e.* ∼5–8 µm) for grains larger than 100 µm, whereas grains smaller than 50 µm in this sample are beyond the detection limit of the current implementation.

(2) Although the CCD detector has significantly better contrast over noise ratio compared with the flat panel, the quality of the resultant grain maps is almost identical except for a marginal improvement in grain indexing for the CCD detector for the sample studied in the present work. This demonstrates a high tolerance of the detector choice for implementing LabDCT on conventional tomography setups.

(3) The reasons for having comparable grain reconstruction quality between the flat panel and the CCD measurements are twofold: First, the quality of spot segmentation is not linearly proportional to the CNR. Second, although the completeness values are lower, the absolute number of spots per grain is higher in the flat panel measurement because it covers a wider range of scattering angles.

(4) Given the superior CNR of the CCD detector, it can be anticipated that further optimization of the acquisition geometry, including source-to-sample distance, pinhole position and dimensions, would have allowed us to overcome the grain size detection limit reported in the current study.

(5) In total, 88% of the grain volume is reconstructed with a very short exposure time of 4 s using the flat panel, opening the possibility of *in situ* LabDCT experiments. With further increases in the exposure time to 20 s, 98% of the grain volume is reconstructed and the total data acquisition time can be confined to less than 1 h.

Given the experimental demonstration with different detectors presented here and open-source reconstruction code (https://gricad-gitlab.univ-grenoble-alpes.fr/TomoX_SIMaP/GrainRecon), 3D grain mapping by LabDCT or other variants using white/pink X-ray or neutron beams will be easier to establish on other instruments and more widely accessible.

## Supplementary Material

Supporting figures. DOI: 10.1107/S1600576723003874/nb5348sup1.pdf

## Figures and Tables

**Figure 12:**
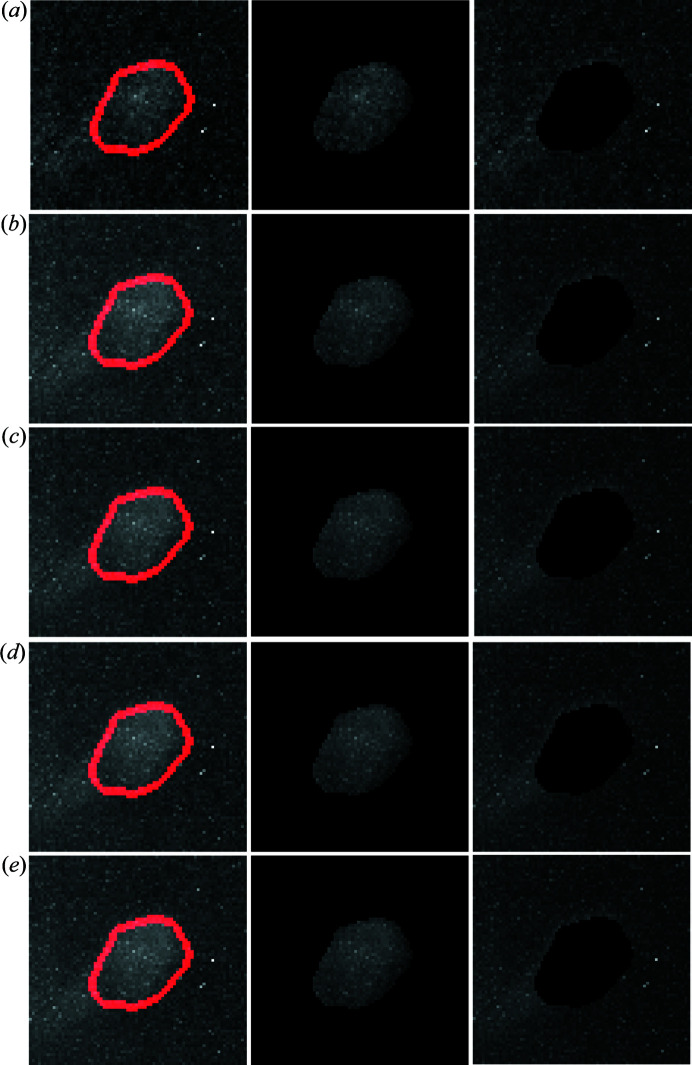
Illustration of CNR determination for the spot 1 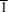 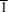 at ω = 288° from grain 25 [see its CNR values for spot 1 in Fig. 7 ▸(*a*)] in images measured by the flat panel detector with an exposure time of (*a*) 4 s, (*b*) 20 s, (*c*) 60 s, (*d*) 120 s and (*e*) 360 s. Left column: a forward spot overlaid onto the experimental image; middle column: region masked by the forward spot to identify the experimental spot; right column: region to determine background noise. Note that another smaller diffraction spot is present at the lower-left corner of the spot of interest, the spots being partially overlapped with each other.

**Table 1:** Experimental parameters for the LabDCT measurements Zoom = 1 + *L*_sd_ / *L*_ss_ characterizes the magnification factor; effective pixel size is calculated as the detector pixel size divided by the zoom; *N*_frame_ is the number of frames.

Experiment	*L*_ss_ (mm)	*L*_sd_ (mm)	Zoom	Pixel size (µm)	Effective pixel size (µm)	*t*_exp_ per frame (s)	*N*_frame_ per angle
CCD	9.2	55.4	7.1	24	3.4	60	6
Flat panel	9.2	224.6	25.4	127	5.0	4	90

**Table 2:** Settings for grain reconstruction parameters that control the indexing and growth

Type	Parameter and symbol	Value	Explanation
Indexing	Minimum completeness, *C*_min_	0.30	Indexing is rejected if *C* < pre-set value
	Maximum acceptable median distance, max*D*_median_	19 pixels	Indexing is rejected if max*D*_median_ > pre-set value

Growth	Distance tolerance of completeness weighted centers, max*D*_center_	3 pixels	Stop updating the center of the grown region if distance ≤ max*D*_center_
	δ_drop-off_	0.02	Accepted into a grown region when 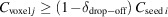

**Table 3:** Geometry parameters before and after the fittings 〈*D*_spot_〉 is computed from ∼2000 spot pairs and the unit is in detector pixels, *i.e.* 24 µm pixel^−1^ for CCD and 127 µm pixel^−1^ for flat panel.

Type	Fitting	*L*_ss_ (mm)	*L*_sd_ (mm)	det*y*0 (mm)	det*z*0 (mm)	φ*_x_* (°)	φ*_y_* (°)	φ*_z_* (°)	〈*D*_spot_〉 (pixels)
CCD	Before	9.16	55.35	−0.35	1.65	0.64	0.36	0.53	6.8
	After	9.21	55.33	−0.40	1.52	−0.02	−0.21	−0.35	5.8

Flat panel	Before	9.16	224.59	0.95	−0.65	0.20	0.23	0.24	6.4
	After	9.04	224.73	0.77	−0.94	0.18	−0.15	0.57	5.7

**Table 4:** Comparison of the number of indexed grains (*N*) between the SR-DCT dataset and the two LabDCT datasets (flat panel and CCD) Grain size 〈*D*〉 is expressed as the mean value and standard deviation. For one-to-multi indexed grains, the number of grains in SR-DCT is given with the paired number of grains in the LabDCT datasets given in brackets. Combined one-to-one and one-to-multi grains are considered as TPs.

Dataset	〈*D*〉 (µm)	*N*						*F*_1_ score
		Total indexed	One-to-one indexed	One-to-multi indexed	FNs		FPs	
					At sample surface	In sample interior		
SR-DCT	101.4±61.7	126	–	–	–	–	–	–
Flat panel	127.4±73.4	79	75	2 (4)	49	0	0	0.759
CCD	122.8±76.6	89	75	7 (14)	44	0	0	0.788

**Table 5:** Indexing comparison for grain reconstructions with different exposure times with respect to SR-DCT *f*_indexed_/*f*_indexed_ (360 s) denotes the ratio of the reconstructed volume with respect to the volume with the longest exposure time of 360 s. Other symbols have the same meanings as in Table 4 ▸.

Dataset	*t*_exp_ (s)	〈*D*〉 (µm)	*N*				*F*_1_ score	*f*_indexed_/*f*_indexed_ (360 s)
			Total indexed	One-to-one indexed	One-to-multi indexed	FNs		
Flat panel	4	146.8±79.9	50	45	3 (6)	78	0.552	0.8854
	20	146.7±73.9	59	56	2 (4)	68	0.630	0.9768
	60	140.7±73.9	65	63	1 (2)	62	0.674	0.9875
	120	141.7±71.1	66	66	0	60	0.688	0.9864
	
CCD	60	144.5±71.6	68	62	3 (6)	61	0.681	0.9774
	120	141.2±73.8	71	69	1 (2)	56	0.714	0.9835

## Appendix A Determination of contrast-to-noise ratios for diffraction spots

CNR is determined by performing a forward projection onto the experimental raw images, as illustrated in Fig. 12 ▸. First, the forward spot is overlaid onto the experimental image and a region of interest is defined as the bounding box of the forward spot oversized by 20 pixels. Then, the region masked by the forward spot is regarded as ‘spot’, whose average pixel value is calculated (  ). The rest of the region is regarded as ‘background’, whose average and standard deviation of the pixel values are calculated (  and  , respectively). Last, CNR is calculated as
