# Supplementary material for: Implementation of grain mapping by diffraction contrast tomography on a conventional laboratory tomography setup with various detectors
Source: J Appl Crystallogr. 2023 May 31;56(Pt 3):810–24. doi: 10.1107/S1600576723003874 (PMC10241044; doi:10.1107/S1600576723003874)
Supplement: Supplementary file 1 [file j-56-00810-sup1.pdf]

JOURNAL OF
APPLIED
CRYSTALLOGRAPHY

Volume 56 (2023)

Supporting information for article:

Implementation of grain mapping by diffraction contrast tomography on a conventional laboratory tomography setup with variable detectors

Haixing Fang, Wolfgang Ludwig and Pierre Lhuissier

Diffraction projections have been acquired on the AlCu alloy sample to study the effects of several key parameters, including sample-to-detector distance (L_{sd}), source voltage and source size, on the quality of the projection, thereby providing a guideline for choosing a proper combination of different experimental parameters for LabDCT experiments using the current tomography instrument. Other parameters, such as sample-to-source distance (L_{ss}), exposure time, pinhole position and size and beamstop used for the CCD and flat panel detectors, were kept constant.

It is concluded that a source voltage of 45 - 60 kV using the nano source in a middle or a large size mode are favorable to obtain high quality LabDCT projections for this sample using the present tomography instrument. Given the same pinhole and L_{ss} as described in the paper, L_{sd} is suggested to be in the range 195 - 250 mm for the flat panel and 50 – 65 mm for the CCD by considering the background noise level, probability of spot overlap and geometric constraints of the instrument. The results are presented as follows together with additional results of LabDCT projections measured with the CCD detector at shorter exposure times compared to the result presented in the paper. Notably, all the images here are shown in log scales to enhance the visibility of diffraction spots in visualizations.

1. Diffraction projection as a function of sample-to-detector distance

For the flat panel, LabDCT diffraction projections were acquired with an exposure time of 4 s and $L_{ss} \approx 9.2$ mm as a function of L_{sd} in the range 375 – 195 mm, with the latter distance close to the geometric constraint of this setup. As shown in Figure S1, the diffraction spots on the images become smaller and at the same time the area affected by high background intensity (caused by scattering & partial transmission from edges of the pinhole) shrinks with decreasing L_{sd}. Visual inspection indicates that the diffraction spots are sharper and more evident from the background at smaller L_{sd}, such as at 225 mm shown in Figure S1c. On the other hand, with decreasing L_{sd} the probability of diffraction spots overlap increases and hence it tends to form a more crowded spot image (see Figure S1d).

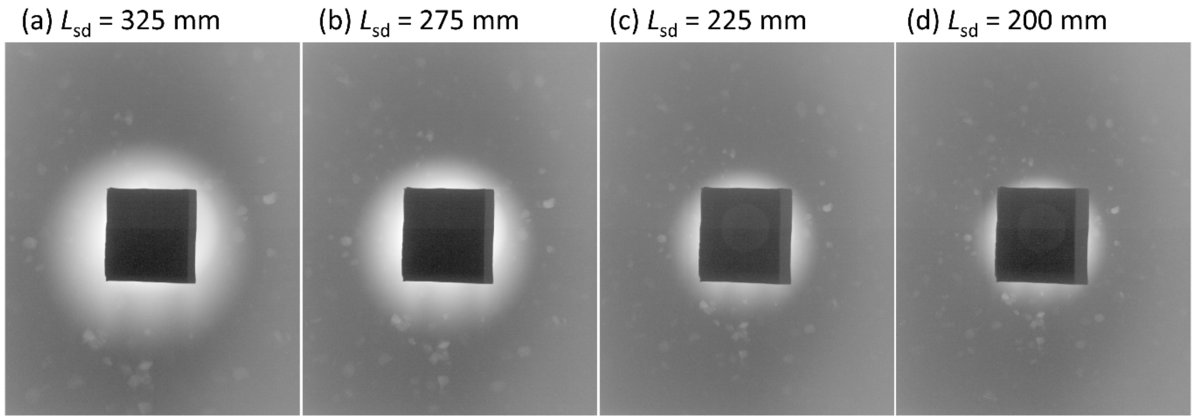

Figure S1. Diffraction projections acquired by the flat panel at a sample-to-detector distance (L_{sd}) of (a) 325, (b) 275, (c) 225 and (d) 200 mm.

Similar result is observed in Figure S2 for the diffraction projections measured with L_{sd} = 95 – 51 mm by the CCD detector with an exposure time of 60 s and L_{ss} ≈ 9.2 mm. The diffraction spots are more magnified at longer L_{sd}, but suffer more harm from the shadow of the direct beam, while at shorter L_{sd} the spots are more distinct from the background but more easily overlapped with others.

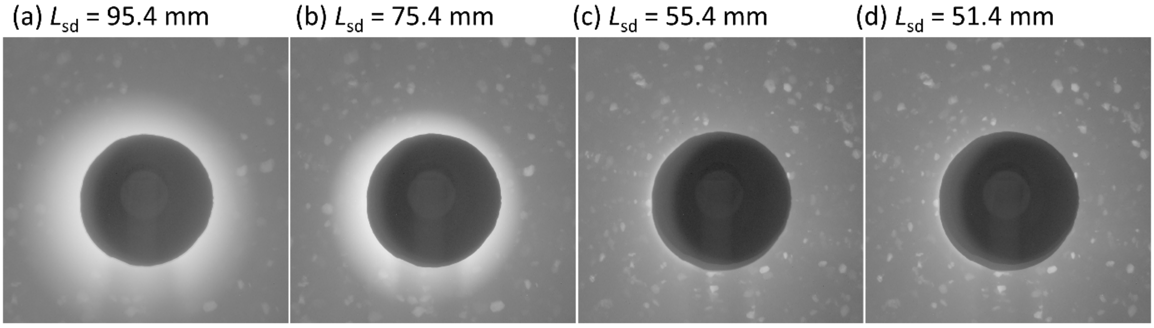

Figure S2. Diffraction projections acquired by the flat panel at a sample-to-detector distance (L_{sd}) of (a) 275, (b) 250, (c) 225 and (d) 200 mm.

To have a quantitative comparison, the average and standard deviation of the background intensity for 8 selected regions in the middle and corner parts of the LabDCT projection (see Figure S3 for their locations) were computed. One may think that the best way to compare the projection quality is to compute the contrast-to-noise ratio of each spot on the projections. However, it should be noted that the same spot across different L_{sd} appears at different locations of the projections (*e.g.* a spot on the corner deems to be less visible than a spot in the middle region because of inhomogeneity in background noise) or even disappears (not able to track the same spot all the time). Therefore, here we only focus on quantifying the background intensities in each region (that are selected to be relatively large to minimize the contribution of the spot intensities) to obtain a guide for evaluating the projection quality.

Figure S4 shows plots of background average intensity ($\bar{I}_{bg}$), standard deviation (σ_{bg}) and their ratio ($\sigma_{bg}/\bar{I}_{bg}$) as a function of L_{sd} for the flat panel. The figure clearly show a significant increase of both $\bar{I}_{bg}$ and σ_{bg} in the middle regions affected by the direct beam at large L_{sd} (Figure S4a and S4b). This leads to strong rise of $\sigma_{bg}/\bar{I}_{bg}$ with increasing L_{sd} for these regions (Figure S4c). Whilst the magnitude is much weaker, the ratios for some corner regions are also increasing with increasing L_{sd}. Figure S4 suggests that keeping L_{sd} = 195 - 250 mm gives a relatively low background intensity as well as a low background noise level.

Figure S5 shows the plots for the CCD projections. The figure also show a dramatic increase of both $\bar{I}_{bg}$, σ_{bg} and $\sigma_{bg}/\bar{I}_{bg}$ in the middle regions with increasing L_{sd}. Although fluctuations of $\sigma_{bg}/\bar{I}_{bg}$ for the corner regions can be seen in Figure S5c, the increase of the ratio becomes evident when L_{sd} > 75 mm. The figure suggests that a distance of L_{sd} = 50 - 65 mm leads to a relatively low background noise level and thus suitable for achieving high quality diffraction projections.

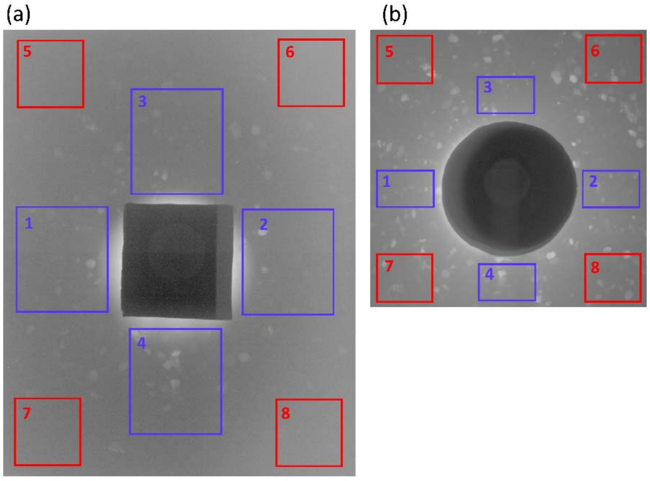

Figure S3. Schematic diagram showing the selection of 8 regions for evaluating the background intensity and noise for (a) flat panel and (b) CCD projections. Regions 1-4 are selected in the middle being symmetric around the beamstop and with a distance of 50 pixels from the beamstop side. Regions 5-8 are selected at four corners with a distance of 50 pixels from each image side. In (a) region 1-4 is 402×554 pixels and region 5-8 is 364×460 pixels; In (b) region 1-4 is 430×297 pixels and region 5-8 is 408×408 pixels.

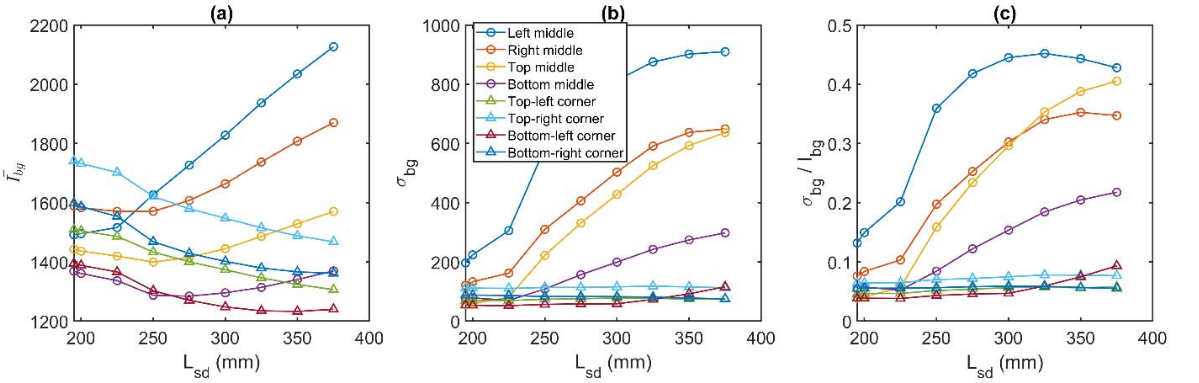

Figure S4. Plots of background average intensity ($\bar{I}_{bg}$), standard deviation (σ_{bg}) and their ratio ($\sigma_{bg}/\bar{I}_{bg}$) as a function of L_{sd} for the LabDCT projections measured by flat panel.

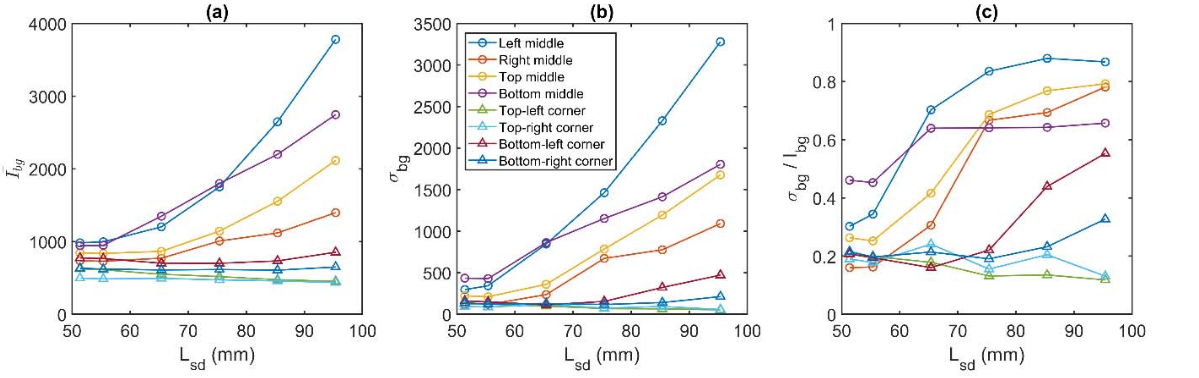

Figure S5. Plots of background average intensity ($\bar{I}_{bg}$), standard deviation (σ_{bg}) and their ratio ($\sigma_{bg}/\bar{I}_{bg}$) as a function of L_{sd} for the LabDCT projections measured by CCD.

2. Diffraction projection as a function of source voltage

LabDCT diffraction projections were acquired using the flat panel with an exposure time of 4 s, $L_{ss} \approx 9.2$ mm, $L_{sd} \approx 225$ mm and nano source in a middle size mode with different source accelerating voltage. Figure S6 shows that the contrast over noise ratio of the diffraction spots decreases with increasing source voltage, especially for the two higher values. As forward simulation (including attenuation, source spectrum and detector efficiency, Fang *et al.*, 2020) indicates that most of the exploitable diffraction signal is expected in the range 15 - 45 keV for this AlCu alloy sample, voltages in the range between 45keV and 60 kV can be considered optimal for the sample studied here.

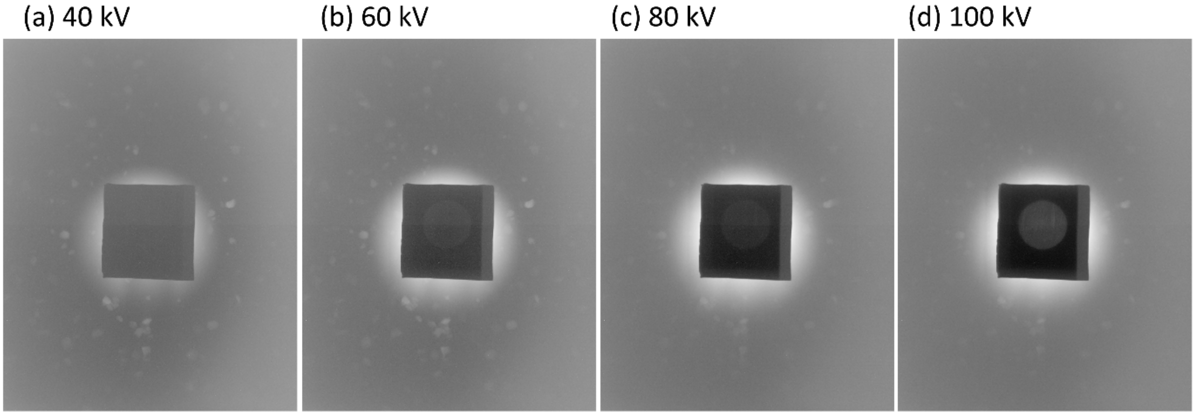

Figure S6. LabDCT projections measured at different source voltages. The current on the target was selected at maximum and was a constant at 30.9 µA for all the voltages.

3. Diffraction projection as a function of source size

Diffraction projections were acquired on the flat panel detector (exposure time 4 s, $L_{ss} \approx 9.2$ mm and $L_{sd} \approx 225$ mm), using different source size modes of the nano source. Figure S7 shows merely visible spots for the small source, corresponding to 10.2 µA current on the anode target (proportional to the total photon flux). The projections look very similar between the middle (30.9 µA) and the large (50.6 µA) source size. Considering a bigger source might lead to geometric blurring of the diffraction spots and thus deteriorate the spatial resolution in the reconstructed grain map, we cautiously chose middle size mode for the tomographic acquisitions presented in the current study. Since no geometric blurring is noticeable in Figure S7, we conclude that some additional improvements may be achieved by performing future experiments in large source mode of the instrument.

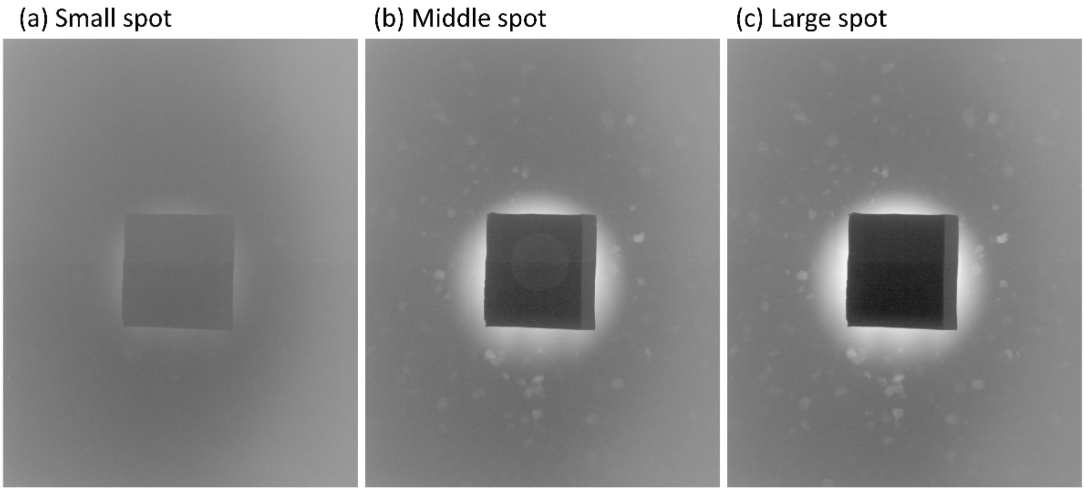

Figure S7. LabDCT projections measured using different spot modes (small, middle and large) of the nano source. The current on the target for the small, middle and large spot is 10.2, 30.9 and 50.6 μA.

4. Diffraction projection as a function of exposure time for the CCD detector

Figure S8 shows LabDCT diffraction projections measured with the CCD detector as a function of exposure time at $L_{ss} \approx 9.2$ mm and $L_{sd} \approx 55.4$ mm (the same distances as listed in Table 1 in the paper). The figure shows very limited number of weak diffraction spots for 4 s (Figure S8a). The number of visible diffraction spots dramatically increases with exposure time increasing to 10 s, for which a high background noise level presents (Figure S8b). However, the diffraction images become smooth and the background noise level does not change significantly when the exposure time increases to 20 s and onwards.

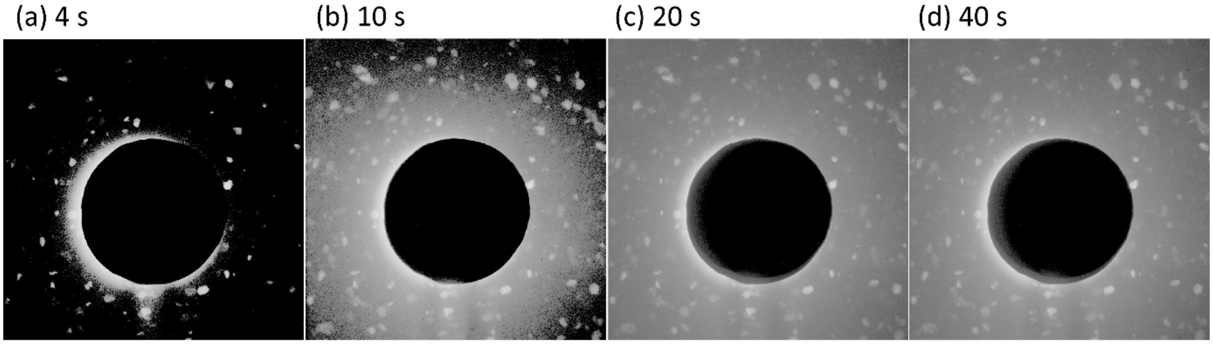

Figure S8. LabDCT projections measured with CCD at different exposure times - 4, 10, 20 and 40 s for (a) - (d), respectively.

References

Fang, H., Juul Jensen, D. & Zhang, Y. (2020). *Acta Cryst. A* **76**, 652-663.
